# Effectiveness of baricitinib in acquired reactive perforating collagenosis: a case report

**DOI:** 10.3389/fimmu.2024.1388274

**Published:** 2024-07-15

**Authors:** Jianfeng Zheng, Yangfeng Ding, Yuanyuan Chen, Yuling Shi, Yunlu Gao

**Affiliations:** ^1^ Department of Dermatology, Shanghai Skin Disease Hospital, Tongji University School of Medicine, Shanghai, China; ^2^ Institue of Psoriasis, Tongji University School of Medicine, Shanghai, China

**Keywords:** acquired reactive perforating collagenosis, baricitinib, case report, diabetes, Th2-type inflammation

## Abstract

**Background:**

Acquired reactive perforating collagenosis (ARPC) poses a clinical challenge with an unclear pathogenesis. This disease has been frequently proven resistant to immunosuppressive treatments, significantly affecting the quality of life of patients. In this report, we highlight the efficacy of baricitinib as a viable option for maintenance therapy in ARPC.

**Case summary:**

An 81-year-old woman presented to our hospital with recurrent pruritus and cup-like ulcerated lesions on her trunk and limbs persisting for 1 year. She exhibited limited response to oral antihistamines and topical steroids. Past medical history revealed a prolonged history of coronary heart disease and type 2 diabetes spanning several years to decades. Histopathological examination revealed cup-shaped depressions filled with necrotic inflammatory debris. In the dermis, a mixed inflammatory infiltrate composed of lymphocytes and histiocytes was observed. Van Gieson staining indicated the elimination of fibrous tissue extending from the dermis into the epidermis. Consequently, a diagnosis of ARPC was established. Due to the inadequate response to conventional treatments and the severe itching, we initiated baricitinib therapy for ARPC, resulting in gradual symptom improvement. Follow-up assessments showed no adverse reactions and normal laboratory findings.

**Conclusion:**

The case report suggests that baricitinib might offer significant therapeutic benefits for ARPC.

## Introduction

Acquired reactive perforating collagenosis (ARPC) is a rare dermatosis often characterized by severe pruritus. First described in 1967 by Mehregan et al. (1), it typically presents as umbilicated hyperkeratotic papules or a dome-shaped lesion with a central crater, frequently affecting the extremities. The Koebner phenomenon has been observed in some patients. In addition, it is commonly associated with diabetes and chronic kidney disease (2). Despite having been recognized for many years, the pathogenesis and etiology of ARPC remain unknown. Moreover, there is a lack of clarity in the treatment consensus for ARPC. In this case report, we present a rare instance of ARPC associated with diabetes, which was effectively managed with baricitinib therapy.

## Case report

**Figure 1:**
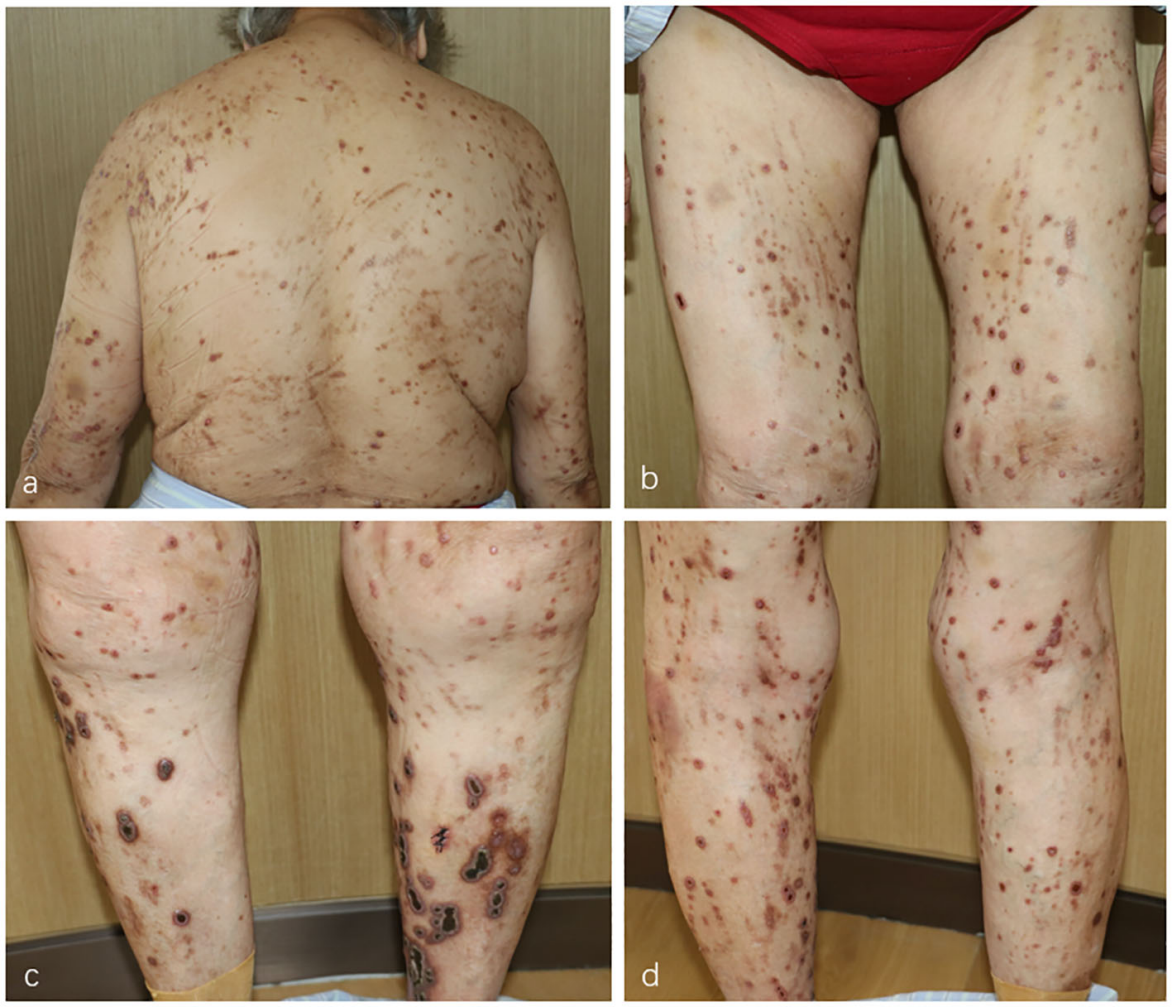
**(A–D)** Multiple umbilicated papules and nodules with a central keratotic crust on the patient’s trunk and limbs.

In September 2023, an 81-year-old woman was referred to the Department of Dermatology with a 1-year history of pruritic skin lesions. These lesions initially presented as umbilicated papules, but progressed to ulcerated plaques, predominantly on the trunk and limbs (**Figure 1**). The Koebner phenomenon was positive as new ulcerated plaques appeared on the normal skin after scratching. She had previously been diagnosed with eczema and had received antihistamines and topical corticosteroids without experiencing improvement in the pruritus and lesions. Her medical history included coronary heart disease for several years and type 2 diabetes mellitus (T2DM) for decades. At present, her T2DM is stable due to insulin treatment.

Upon admission, a series of serological tests, including acquired immunodeficiency syndrome (AIDS) screening; rapid plasma reagin test; hepatitis B virus serological markers; hepatitis C virus antibodies; T-SPOT; liver and kidney function tests; whole blood cell test; and C-reactive protein, anti-streptolysin “O,” antinuclear antibody, extractable nuclear antigen, and antineutrophil cytoplasmic antibody tests, yielded negative results, except for fasting blood glucose (7.89 mmol/L), glycosylated hemoglobin (8.5%), erythrocyte sedimentation (27 mm/h), and total IgE (682.6 kU/L).

**Figure 2:**
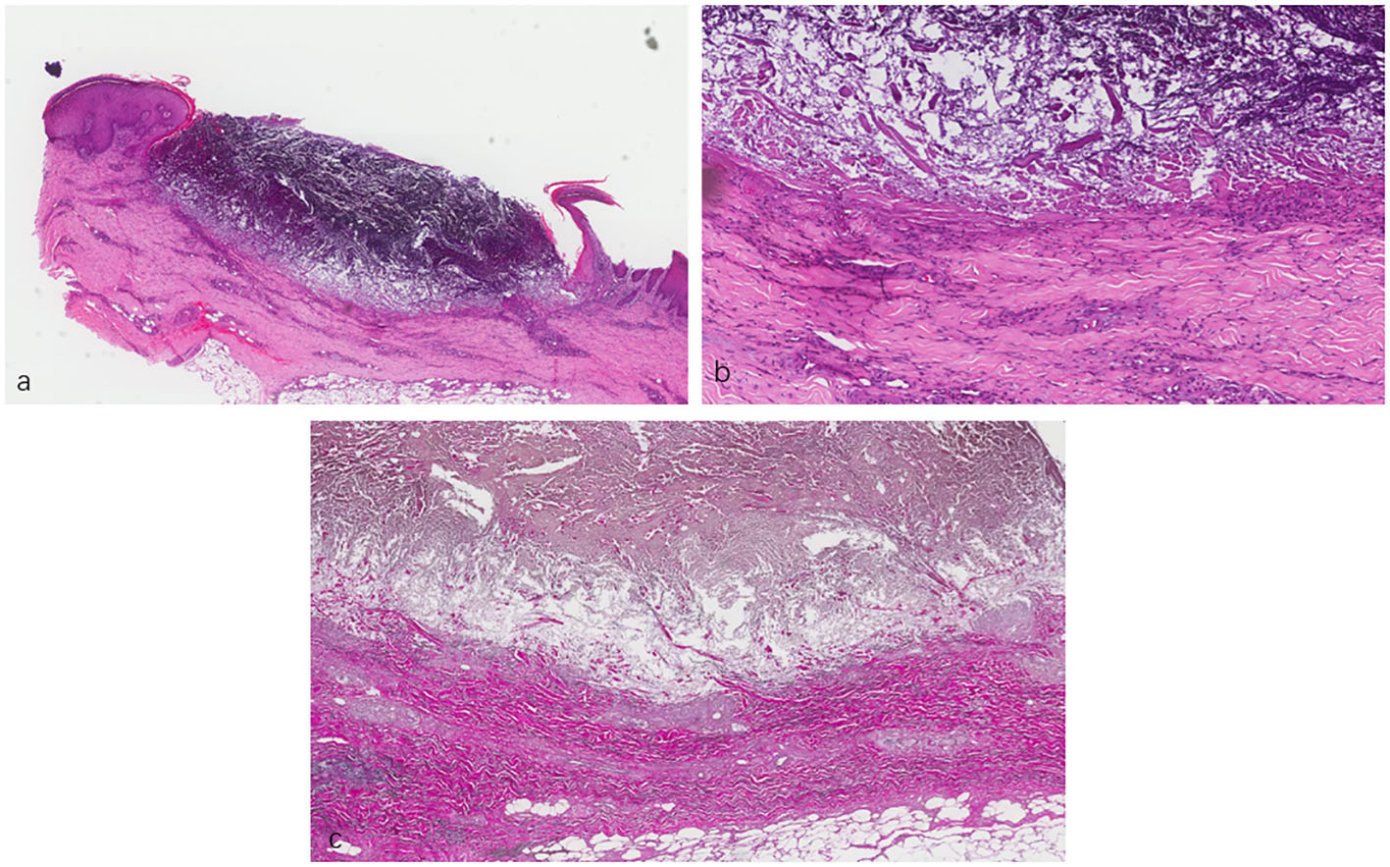
**(A, B)** Lesional skin biopsy showing a cup-shaped depression plugged with necrotic inflammatory debris. **(C)** Van Gieson staining showing elimination of fibrous tissue through the dermis into the epidermis. **(A)** ×40. **(B)** ×200. **(C)** ×400.

A skin biopsy was subsequently performed, with the results of hematoxylin and eosin staining revealing a cup-shaped depression filled with necrotic inflammatory debris and a mixed inflammatory infiltrate composed of lymphocytes and histiocytes in the dermis. Elastica van Gieson staining showed fibrous tissue being ejected through the epidermis (**Figure 2**). In line with the clinical findings, a diagnosis of ARPC was considered. On the one hand, the patient’s itching was severe [Numeric Rating Scale (NRS) score of 9]; on the other hand, dupilumab did not achieve the expected effect in our other cases. Therefore, we initiated a therapeutic challenge with oral baricitinib at 2 mg QD. At 10 days after treatment, there was no significant improvement in her eruptions and itching. However, we recommended that the patient continue with the baricitinib regimen.

**Figure 3:**
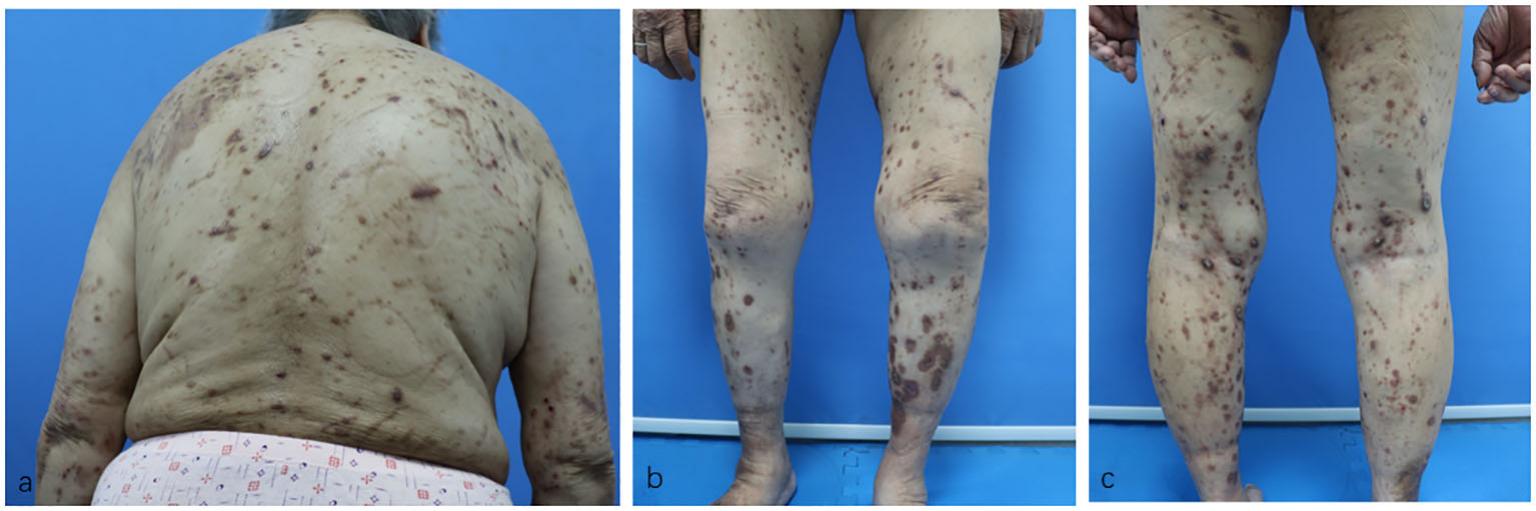
**(A–C)** Residual hyperpigmented lesions, atrophic scars, and a few superficial erosions on the patient’s trunk and limbs after baricitinib treatment.

**Figure 4:**
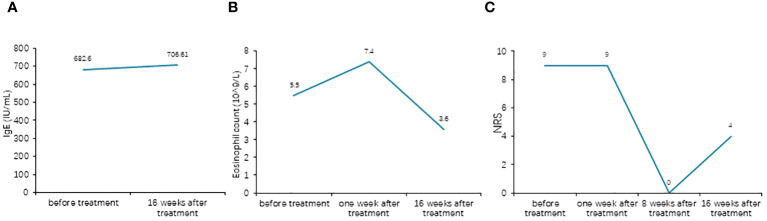
**(A)** Comparison of the total IgE levels before and after treatment. **(B)** Comparison of the eosinophil counts before and after treatment. **(C)** Comparison of the Numeric Rating Scale (NRS) scores before and after treatment.

Surprisingly, her eruptions and itching showed complete improvement by the eighth week. At this point, the patient decided to discontinue the baricitinib treatment. Unfortunately, her lesions and itching gradually reappeared after discontinuation, prompting her to revisit the Department of Dermatology on December 28, 2023. During the physical examination, cup-like ulcerated lesions on her trunk and limbs were noted, along with multiple scar formations (**Figure 3**). The NRS score increased to 4 (**Figure 4**). Laboratory examinations during the second admission revealed negative results for the liver and kidney function tests, whole blood cell tests, and C-reactive protein, except for fasting blood glucose (15.54 mmol/L), glycosylated hemoglobin (10.2%), and total IgE (706.61 kU/L). With the patient’s consent, we resumed maintenance therapy with baricitinib at 2 mg QD. In April 2024, we conducted our last telephone follow-up of the patient, during which her eruptions and itching showed complete improvement.

## Discussion

ARPC is most frequently documented with concomitant T2DM and chronic kidney disease, as well as other systemic pathologies including malignancies, hypothyroidism, liver disorders, neurodermatitis, AIDS, and malignant hypertension (3). The giant variant typically presents as large (>1 cm) crateriform lesions. Central adherent keratotic plugs, severe pruritus, and erythematous halos are characteristics of this condition. Our patient exhibited a typical presentation of the “giant” variant of this disease, forming lesions with the presence of crusted plaques and central keratin plugs. Lesions often develop on the trunk or the limbs, areas easily accessible by hand (4). However, the treatment of perforating dermatoses is challenging. There is currently no consensus on treatment. Several therapeutic options have been reported as first-line therapy in ARPC, including topical corticoids, local and systemic retinoids, and UVB phototherapy, with variable results. Dynamic phototherapy, amitriptyline, and doxycycline have also been the subject of some studies (5, 6).

The etiology of ARPC is unknown; however, many theories have been posited for ARPC, tying superficial trauma of the skin to the elimination of biochemically altered type IV collagen fibers from the basement membrane. An early proposed mechanism for ARPC hypothesized that microcrystal-like deposits in the dermis biochemically altered the collagen fibers. Scratching then causes microtrauma, leading to the degradation of the basement membrane and the transepidermal elimination of collagen fibers (7). Furthermore, altered collagen may be induced by hypoxic states due to the vessel wall thickening associated with diabetic microvasculopathy. Allopurinol, a xanthine oxidase inhibitor, might decrease the elevated uric acid levels correlated with T2DM, reducing the crystal deposits within the dermis. However, recent studies have observed enhanced dermal infiltration of CD3^+^ T cells with a predominance of T helper 2 (Th2) cells in ARPC, similar to atopic dermatitis (AD). In addition, the cytokines interleukin 4 (IL-4) and IL-13, which act on neurons to promote itching, were also significantly upregulated in ARPC (8). Moreover, there is now an increasing amount of clinical data that support the use of dupilumab in ARPC (8, 9). These findings suggest that Th2-type inflammation is involved in the pathogenesis of ARPC. Our patient was successfully treated with a Janus kinase (JAK) inhibitor.

JAK inhibitors are a novel class of oral immunosuppressant medications licensed for the management of AD, psoriasis, rheumatoid arthritis, and alopecia areata. By inhibiting the action of four key tyrosine kinases, i.e., JAK1, JAK2, JAK3, and tyrosine kinase 2 (TYK2), they affect various cell lineages, including CD8-positive T cells, and suppress the interferon gamma and interleukin pathways (10). Baricitinib is a JAK1/JAK2 inhibitor used to treat severe rheumatoid arthritis, alopecia areata, and AD (11–13). More widespread cytokine perturbation via JAK inhibition is beneficial when the disease pathogenesis is complex and not well understood (14). In our case, baricitinib was significantly more effective than the conventional treatment for ARPC. Baricitinib reduced the lesion size and improved pruritus within 4 weeks of treatment. After 8 weeks of treatment, the patient achieved clearance of skin lesions and relief from pruritus, with no adverse events (AEs).

## Conclusion

To the best of our knowledge, this case marks the inaugural utilization of baricitinib in the treatment of ARPC. This case underscores the efficacy of baricitinib for ARPC and advocates its consideration in instances of conventional treatment-resistant ARPC. While certain studies have indicated the involvement of type 2 inflammation in the pathogenesis of ARPC, further clinical and foundational research is imperative to corroborate this conclusion.

## Data availability statement

The original contributions presented in the study are included in the article/supplementary material. Further inquiries can be directed to the corresponding authors.

## Ethics statement

Written informed consent was obtained from the individual(s) for the publication of any potentially identifiable images or data included in this article.

## Author contributions

JZ: Data curation, Formal Analysis, Investigation, Writing – original draft, Writing – review & editing. YD: Resources, Writing – review & editing. YC: Data curation, Resources, Writing – review & editing. YS: Project administration, Resources, Writing – review & editing. YG: Project administration, Resources, Supervision, Validation, Writing – review & editing.
